# Account of Diseases in an Eastern District of London, from the 20th of January to the 20th of February, 1801

**Published:** 1801-03

**Authors:** 


*Account of Difeafes in an Eaftern Diftrict of London, from the 20th of January to the 20th of February,* 1801.

| | No. of Cafes. | | No. of Cafes. |
|---|---|---|---|
| ACUTE DISEASES. | | Hyfteria - - - - - - - | 4 |
| Typhus - - - - - - | 17 | Cephalæa - - - - - - | 7 |
| Pneumonia - - - - - - | 4 | Hemiplegia - - - - - | 2 |
| Eryfpielas - - - - - - | 1 | Vertigo - - - - - - - | 5 |
| Cynanche Tonfillaris - - | 2 | Fluor Albus - - - - | 6 |
| Dyfenteria - - - - - | 2 | Dyfuria - - - - - - | 4 |
| CHRONIC DISEASES. | | Lumbago - - - - - - | 2 |
| Cough - - - - - - - | 20 | Rheumatifmus - - - - | 23 |
| Dyfpnœa - - - - - - | 12 | PUERPERAL DISEASES. | |
| Cough and Dyfpnœa - | 15 | Low Puerperal Fever - - | 3 |
| Catarrhus - - - - - - | 4 | Dolores Poft Partum - - | 5 |
| Hæmoptyfis - - - - - | 3 | Maftodynia - - - - - - | 4 |
| Hydrothorax - - - - - | 5 | Menorrhagia Lochialis - | 6 |
| Afcites - - - - - - - | 2 | INFANTILE DISEASES. | |
| Anafarca - - - - - - | 7 | Aphthæ - - - - - - - | 7 |
| Gaftrodynia - - - - - | 15 | Diarrhœa - - - - - - | 12 |
| Enterodynia - - - - - | 17 | Dentition - - - - - - | 3 |
| Diarrhœa - - - - - - | 20 | | |

It will appear from the annexed Lift, that a fever, which has for fome time been confidered as the prevailing epidemic, ftill continues to hold a diftinguifhed place in the catalogue of difeafes. It has been repeatedly remarked, that a great determination to the head forms a diftinguifhing characteriftic of this fever. Affections of the head have appeared under different forms, and in various degrees; in fome of the cafes there has been fo fierce a delirium, as to give the difeafe the appearance of phrenitis: The mode of its approach alfo, was frequently fuch as to indicate a difeafed ftate of the brain; and fo great was the torpor of the mental faculties, that it was difficult to roufe the attention to any object, or when roufed, to engage it for a fufficient length of time to anfwer any purpofe. After a reply to any queftion, which was made with a degree of quicknefs, the patient returned to a ftate of fullen inattention, and the countenance indicated difpleafure at being taken notice of, or roufed to any exertions. Thefe fymptoms bearing a near refemblance to thofe which frequently occur previoufly to an attack of mania, naturally produced an alarm refpecting the future ftate of the patient's mind; but being fucceeded by by more evident ſymptoms of febrile action, this apprehenſion gradually ſubſided. In ſome caſes the returns of furious paroxyſm were ſo frequent and ſo violent, that it became neceſſary to place the patient under reſtraint, and a ſtraight waiſtcoat was found a convenient mode of preventing his doing miſchief to himſelf, or his attendants. In two or three of the caſes referred to, an eruption appeared on different parts of the body, very much reſembling that of the meaſles, but without producing any particular change in the ſtate of the diſeaſe. In one of the caſes which proved fatal, there was a conſiderable rigidity of the muſcles of the jaw, ſo that it was with difficulty that the neceſſary food, or medicine, were conveyed to the ſtomach.

It may be hoped that the change which has lately taken place in the temperature of the atmoſphere, will produce a favourable change in the ſtate of this diſeaſe: On the other hand, we have reaſon to fear, that it will occaſion a leſs pleaſant alteration in the ſtate of pneumonic diſeaſes, which begin already to increaſe in their number, and to be attended by an aggravation of ſymptoms.

